# Supplementary figures and images for: Implications of gene tree heterogeneity on downstream phylogenetic analyses: A case study employing the Fair Proportion index
Source: PLoS One. 2024 Apr 25;19(4):e0300900. doi: 10.1371/journal.pone.0300900 (PMC11045071; doi:10.1371/journal.pone.0300900)

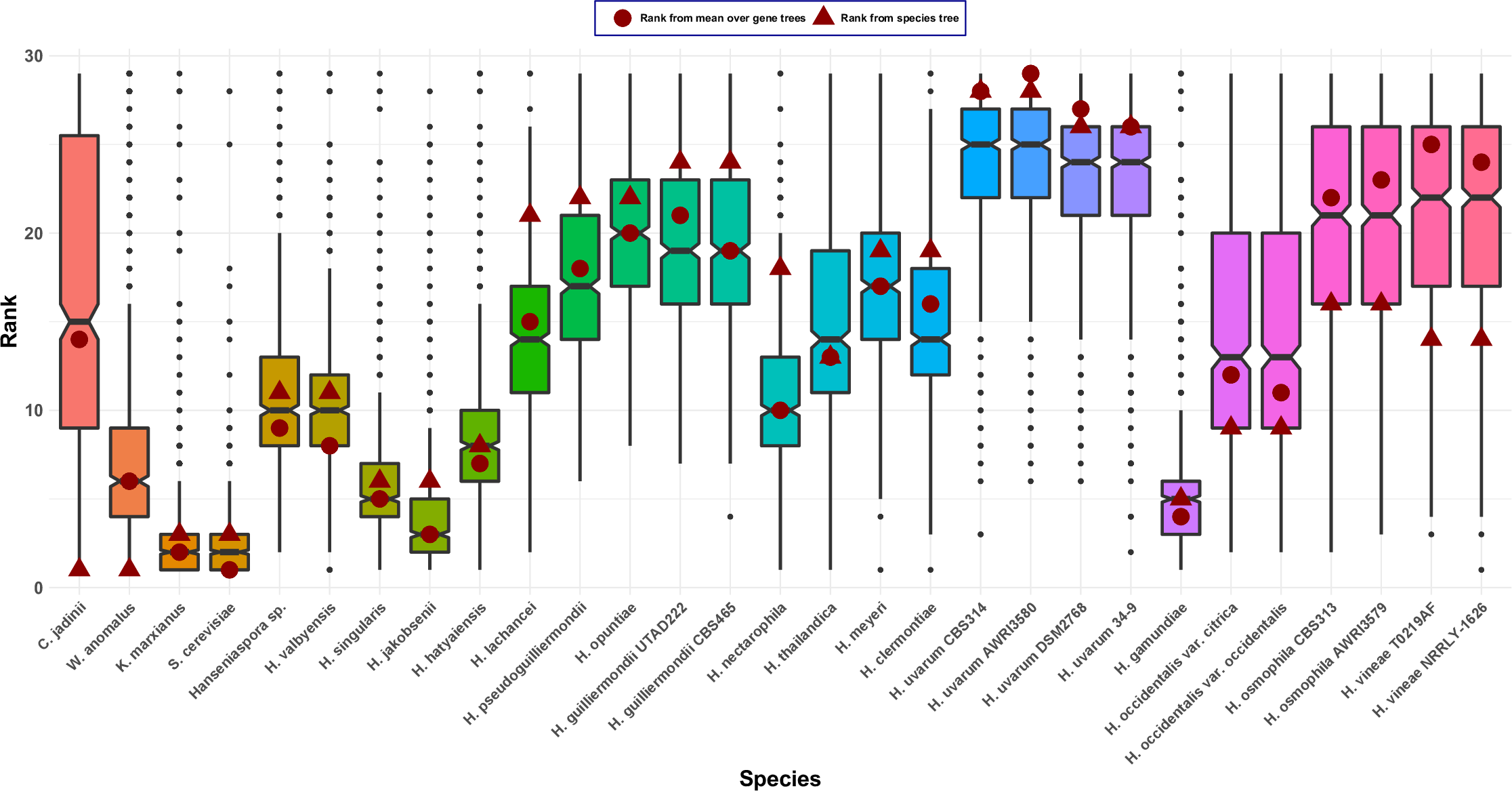

Supplement: S1 Fig — In addition, the ranks obtained from the average FP index across the 683 gene trees (dots) and the ranks on the species tree (triangles) are depicted. (TIF) [file pone.0300900.s001.tif]

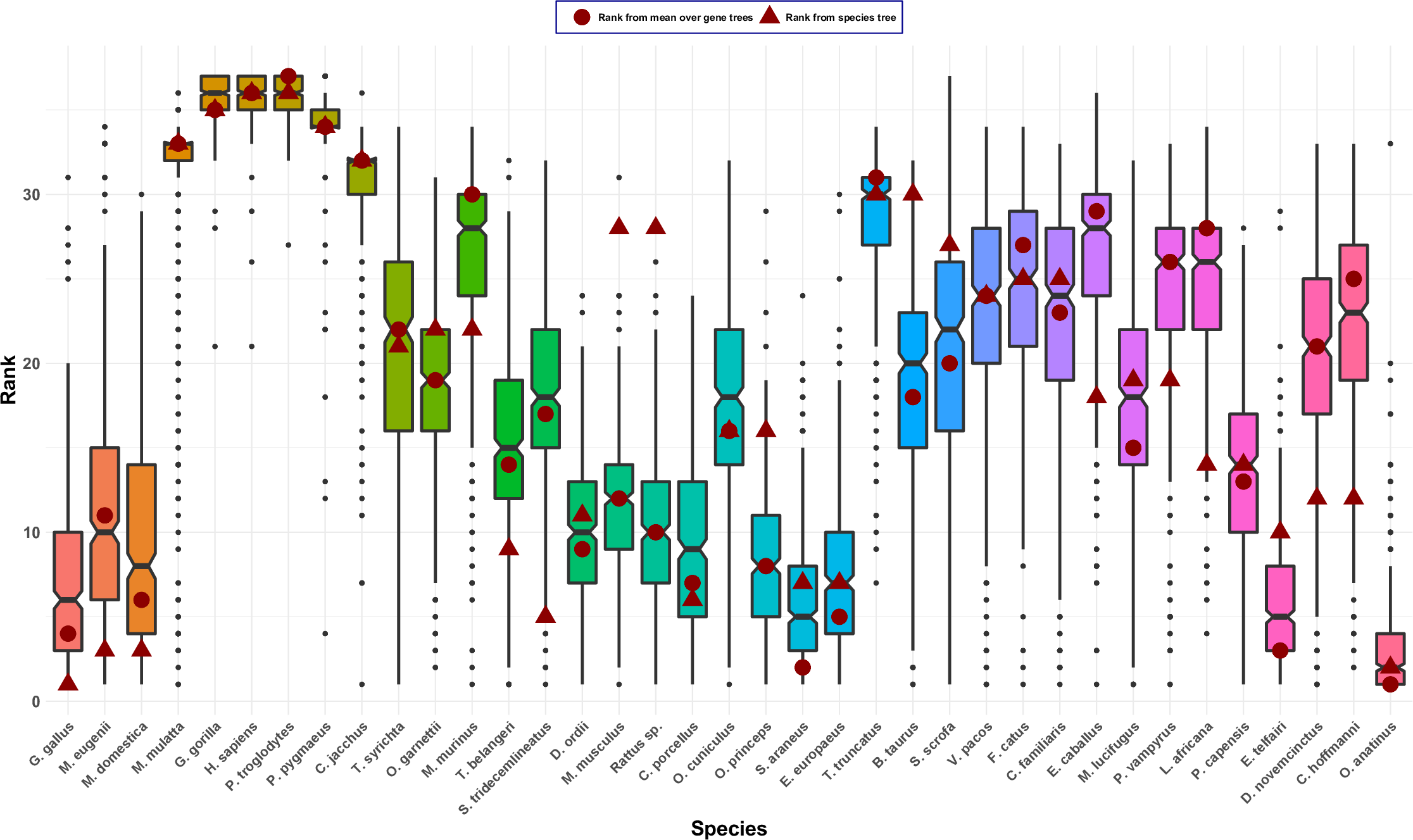

Supplement: S2 Fig — In addition, the ranks obtained from the average FP index across the 447 gene trees (dots) and the ranks on the species tree (triangles) are depicted. (TIF) [file pone.0300900.s002.tif]

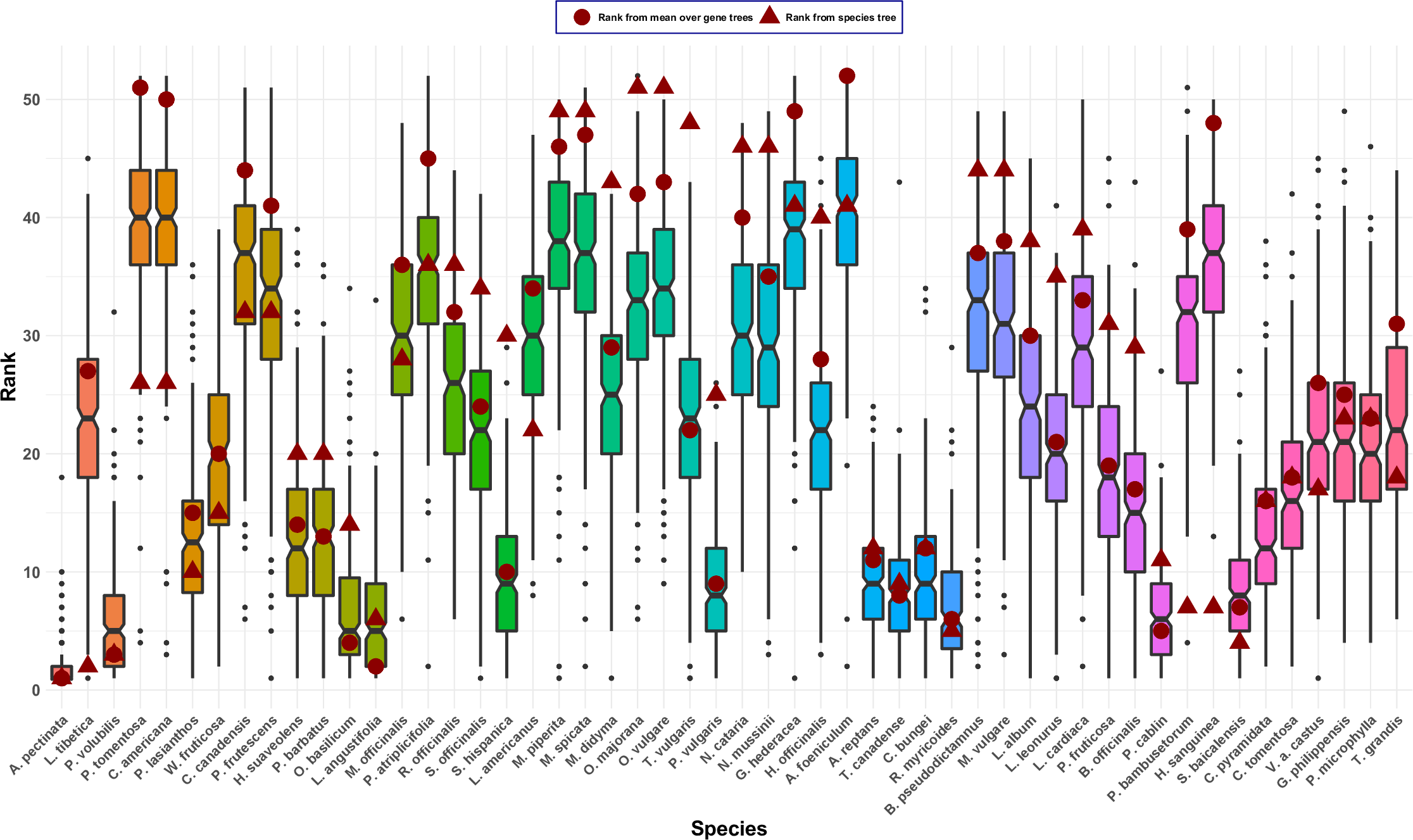

Supplement: S3 Fig — In addition, the ranks obtained from the average FP index across the 318 gene trees (dots) and the ranks on the species tree (triangles) are depicted. (TIF) [file pone.0300900.s003.tif]

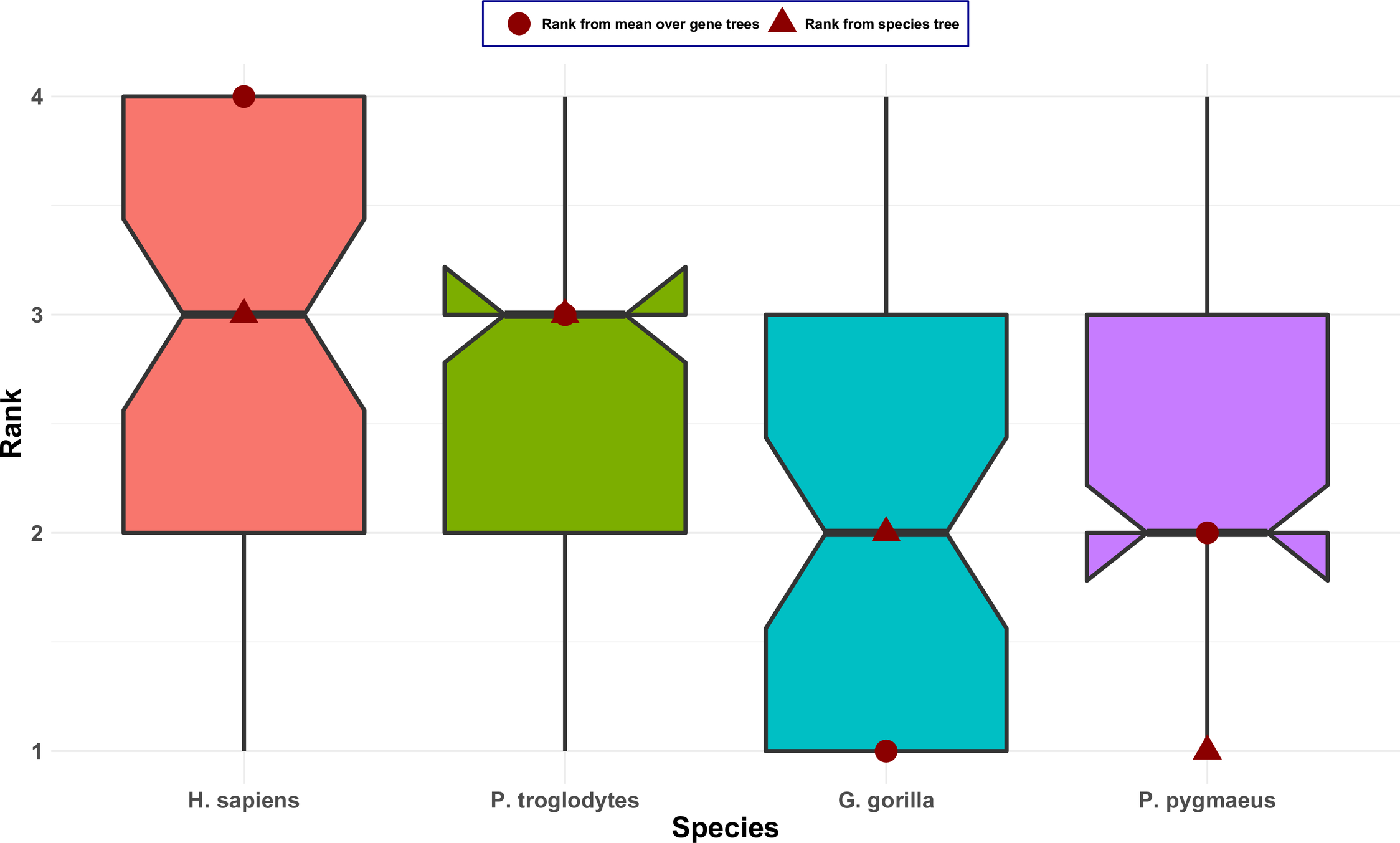

Supplement: S4 Fig — In addition, the ranks obtained from the average FP index across the 52 gene trees (dots) and the ranks on the species tree (triangles) are depicted. (TIF) [file pone.0300900.s004.tif]

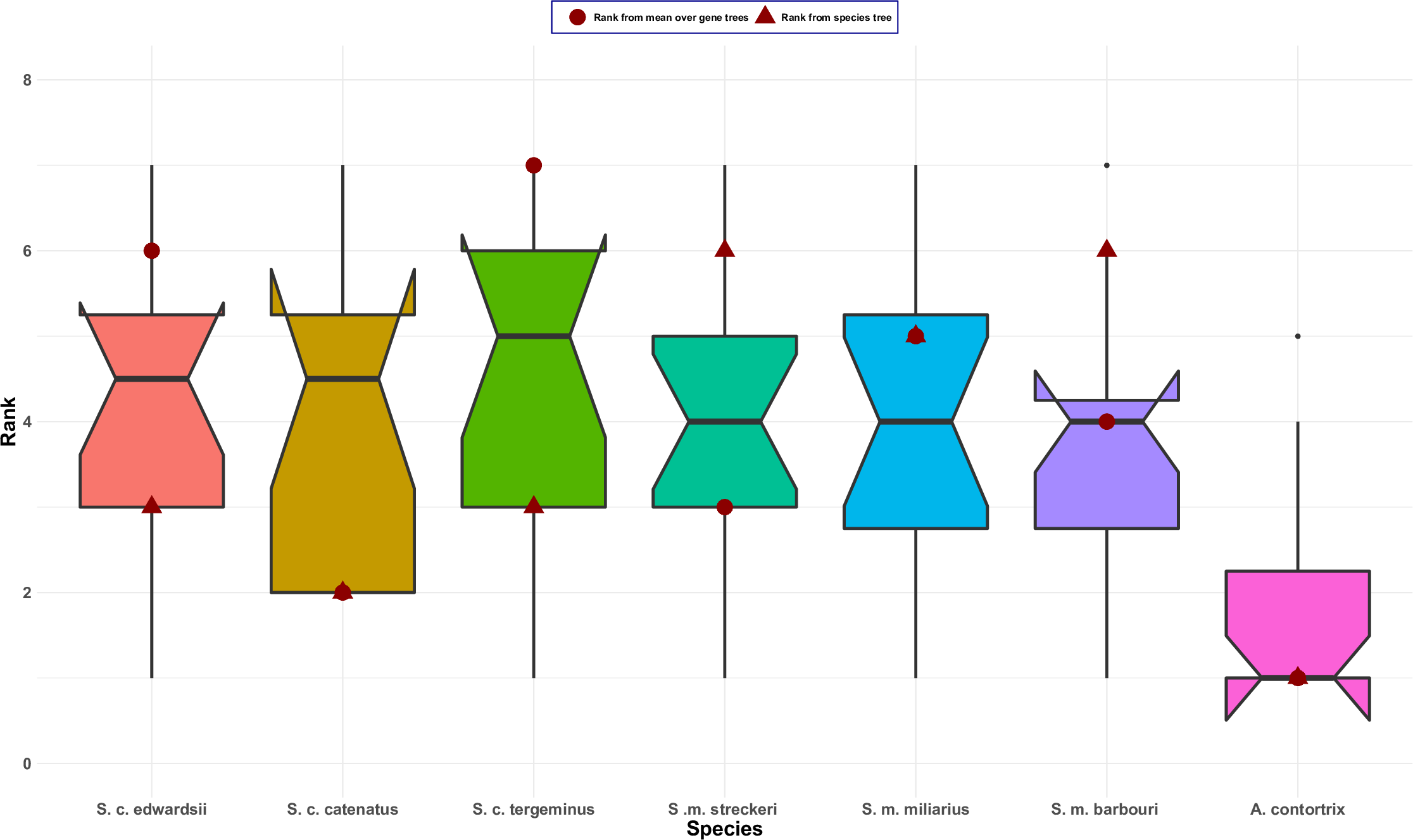

Supplement: S5 Fig — In addition, the ranks obtained from the average FP index across the 16 gene trees (dots) and the ranks on the species tree (triangles) are depicted. (TIF) [file pone.0300900.s005.tif]

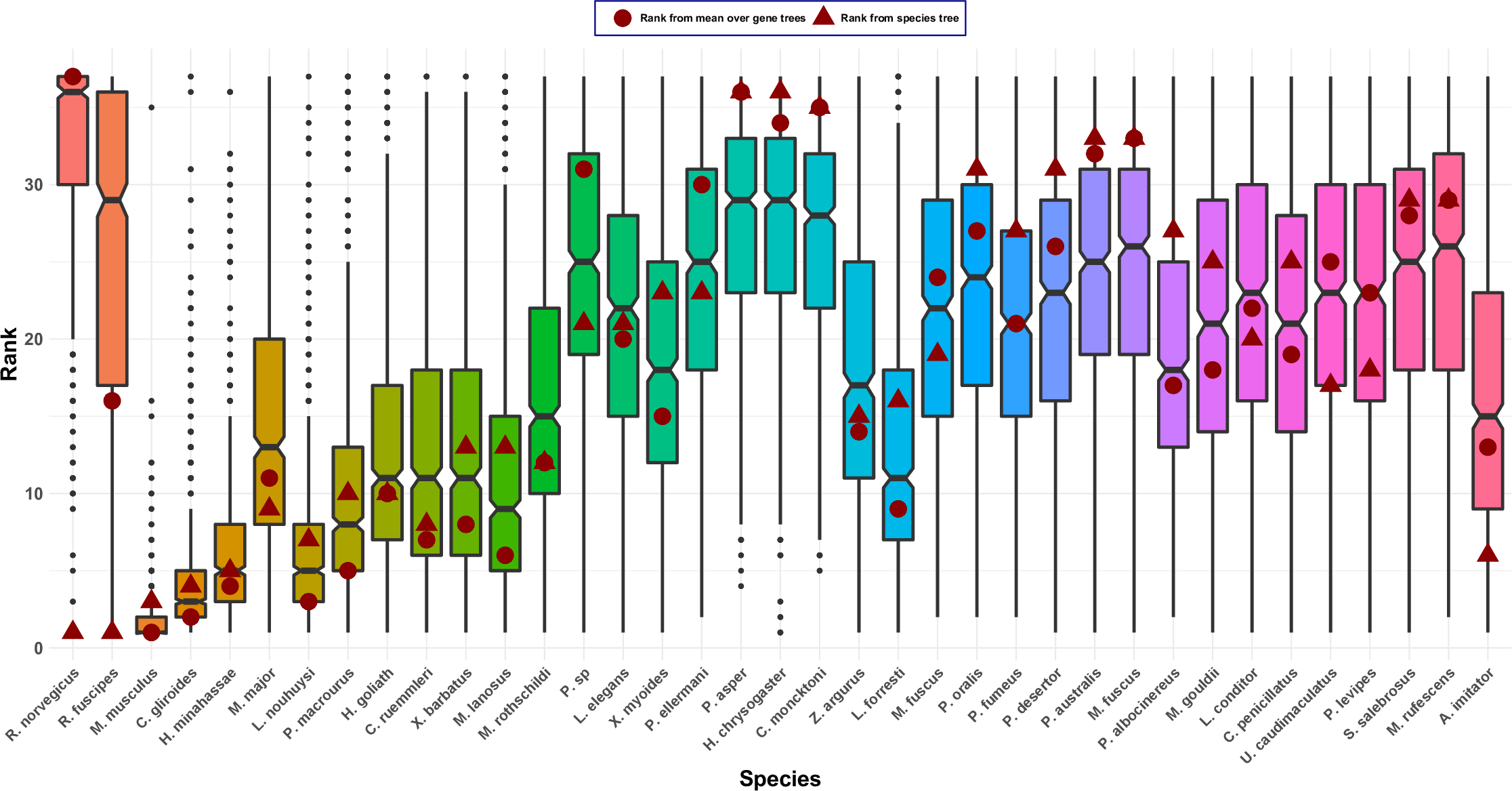

Supplement: S6 Fig — In addition, the ranks obtained from the average FP index across the 761 gene trees (dots) and the ranks on the species tree (triangles) are depicted. (TIF) [file pone.0300900.s006.tif]

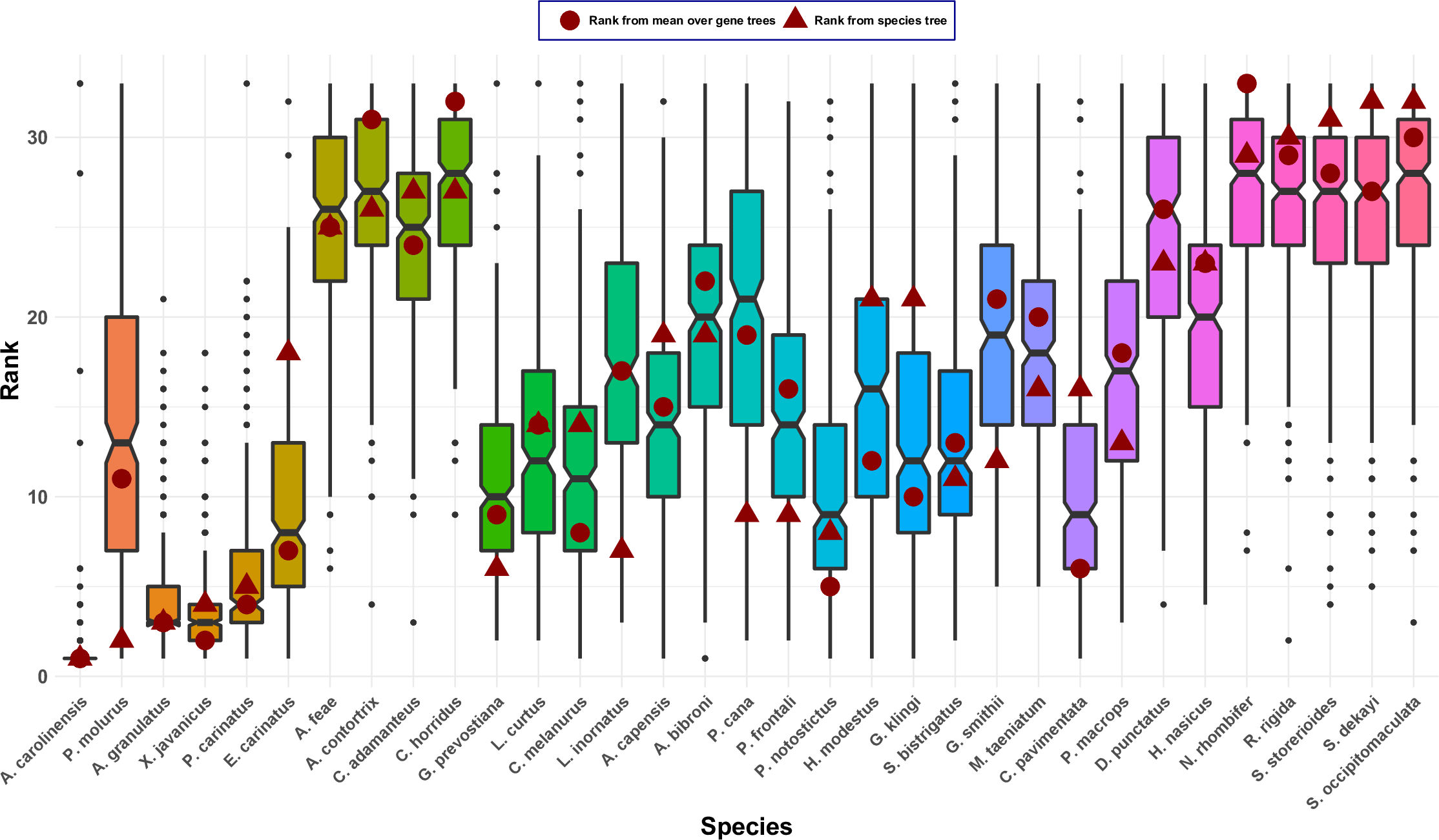

Supplement: S7 Fig — In addition, the ranks obtained from the average FP index across the 333 gene trees (dots) and the ranks on the species tree (triangles) are depicted. (TIF) [file pone.0300900.s007.tif]

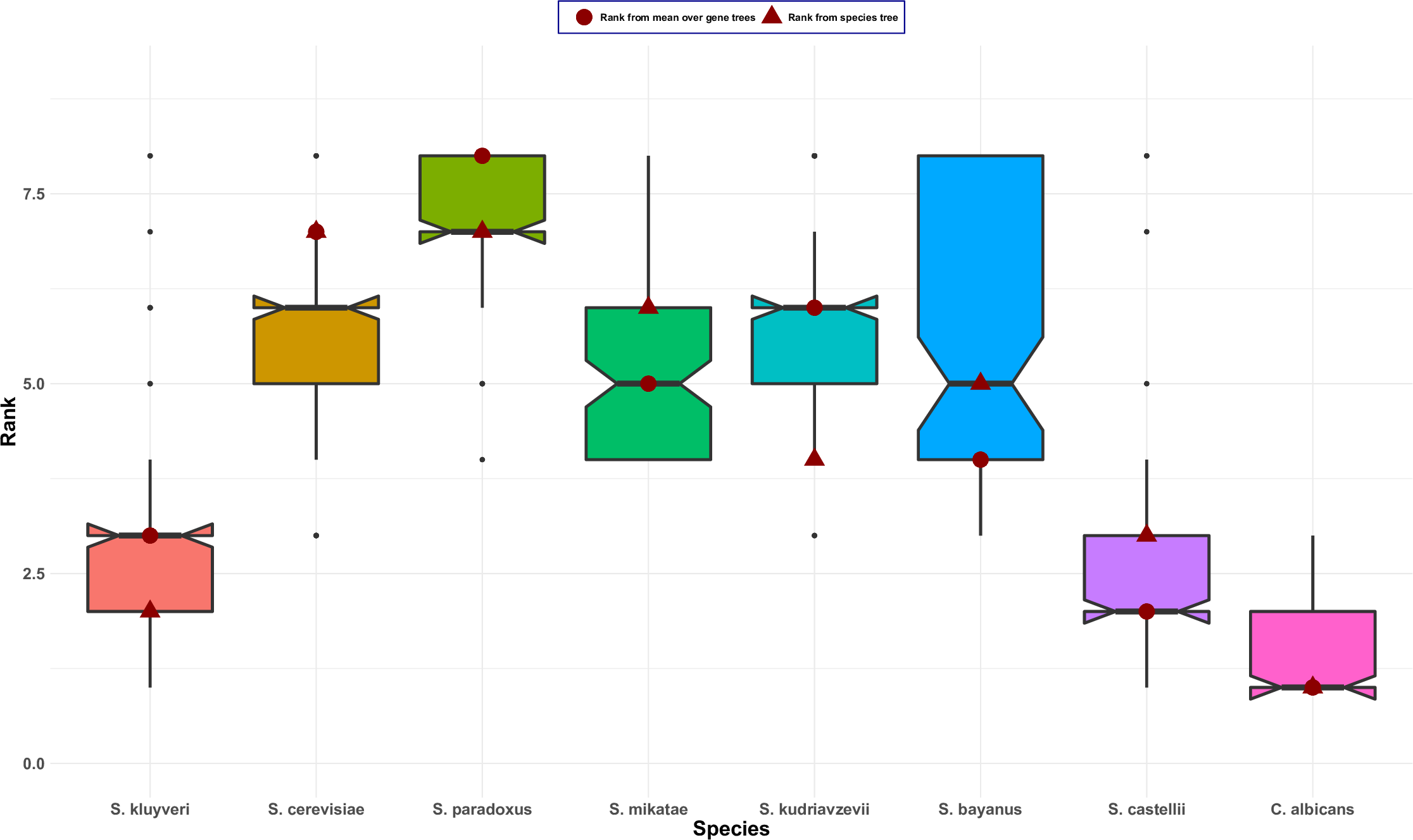

Supplement: S8 Fig — In addition, the ranks obtained from the average FP index across the 106 gene trees (dots) and the ranks on the species tree (triangles) are depicted. (TIF) [file pone.0300900.s008.tif]
