# Supplementary material for: Implications of gene tree heterogeneity on downstream phylogenetic analyses: A case study employing the Fair Proportion index
Source: PLoS One. 2024 Apr 25;19(4):e0300900. doi: 10.1371/journal.pone.0300900 (PMC11045071; doi:10.1371/journal.pone.0300900)
Supplement: S1 Appendix — The Appendix provides details of the methods used to curate each of the nine data sets included in the manuscript. (PDF) [file pone.0300900.s009.pdf]

# Appendix for
# "Implications of gene tree heterogeneity on downstream phylogenetic analyses: A case study employing the Fair Proportion index"

Kristina Wicke, Md Rejuan Haque, and Laura Kubatko

## 1 Details about the 9 data sets

In the following, we describe in more detail how the 9 multilocus data sets used in this study were processed prior to computing the Fair Proportion index.

**Dolphin data set.** This data set obtained from [1] contained DNA sequence data from 24 genes for 47 aquatic mammals. Due to large amounts of missing data, we reduced it to a "complete" data set by excluding 19 species (the species excluded were *D. tropicalis*, *S. frontalis*, *S. clymene*, *S. guianensis*, *L. peronii*, *L. cruciger*, *L. australis*, *C. hectori*, *C. heavisidii*, *C. eutropia*, *G. melas*, *Or_ breviostris*, *Ph_ dalli*, *I. geoffrensis*, *P. blainvillei*, *L. vexillifer*, *Z. cavirostris*, *P. minor*, and *M. novaeangliae*) and 2 genes (the genes excluded were STAT5 and TH), resulting in a total of 22 genes and 28 species. We then estimated gene trees under the GTR+Gamma model and with *P. macroce* as the outgroup using RAxML version 8.2.12 [2]. A species tree estimate was obtained using SVDquartets and maximum likelihood branch lengths were computed under the GTR+Gamma model and enforcing a molecular clock in PAUP* (again using *Ph. macroce* as the outgroup).

**Fungi data set.** This data set was originally collected by [3] and re-analyzed by [4]. We downloaded the data set from [4] (`https://datadryad.org/stash/dataset/doi:10.5061%2Fdryad.9p8cz8wc5`) and used the data found in the directory
`2_Shen_et_al_Empirical_datasets/2_fungi/removal_of_inconsistent_genes/ASTRAL` consisting of gene and species tree estimates based on 683 genes for a total of 25 individuals/variants of 18 bipolar budding yeasts and four outgroups. In order to be able to directly use the authors' estimated gene and species trees, we included all individuals in the analysis, even if they came from the same species. However, we re-computed maximum likelihood branch lengths for the species tree under the LG model (using the full amino acid sequence alignment found in file `29taxa_683genes_ML2ASTRAL_CCC.fas` in the directory `2_Shen_et_al_Empirical_datasets/2_fungi/removal_of_inconsistent_genes/Concatenation_ML`) and enforcing a molecular clock in PAUP*. Both gene and species trees were rooted using species *C. jadinii* as the outgroup.

*Corresponding author
*Email address:* `kubatko.2@.osu.edu`

**Mammal data set.** This data set was originally collated by [5] and re-analyzed by [6]. We obtained the DNA sequence data from [5] (`https://datadryad.org/stash/dataset/doi:10.5061%2Fdryad.3629v`) and gene tree estimates based on 447 genes for 33 species of mammals and 4 outgroup species from [6], and directly used the published gene trees (rooting them using *G. gallus* as the outgroup) for our analysis. A species tree estimate was obtained using SVDquartets, and maximum likelihood branch lengths for the species tree were computed under the GTR+Gamma model and enforcing a molecular clock in PAUP*.

**Plant data set.** This data set was originally collated by [7] and re-analyzed by [4]. We downloaded the data set from [4] (`https://datadryad.org/stash/dataset/doi:10.5061%2Fdryad.9p8cz8wc5`) and used the data found in the directory `2_Shen_et_al_Empirical_datasets/3_plant/removal_of_inconsistent_genes` consisting of DNA sequence data as well as gene and species tree estimates based on 363 genes for 48 Lamiaceae and 4 outgroup species. We chose taxon *L. tibetica* as the outgroup and rooted the gene trees provided in the subfolder `ASTRAL`. However, 45 of them (corresponding to the following lines in the file `52taxa_363genes_ML2ASTRAL_CCC.trees`: 10, 28, 29, 34, 43, 49, 50, 52, 53, 58, 62, 63, 79, 95, 116, 123, 131, 144, 150, 155, 170, 188, 202, 208, 216, 222, 229, 239, 245, 256, 259, 273, 274, 278, 281, 296, 307, 309, 311, 312, 338, 343, 346, 354, 362) did not contain this outgroup species and were discarded, resulting in a total of 318 gene trees. We remark, however, that not all of the remaining 318 gene trees contained all 52 taxa, i.e., there was a certain amount of missing data. Moreover, we excluded the sequence data corresponding to the 45 removed genes from the full alignment (file `3_plant_52taxa_363genes_ML2ASTRAL_CCC.fas` found in the subfolder `Concatenation_ML`) and estimated a species tree based on the remaining 318 genes using SVDquartets. Finally, maximum likelihood branch lengths were computed under the GTR+Gamma model and enforcing a molecular clock in PAUP*.

**Primate data set.** This data set obtained from [8] contained DNA sequence data from 52 genes for four primate species. We estimated gene trees under the GTR+Gamma model and a species tree using RAxML version 8.2.12, respectively SVDquartets. In both cases, trees were rooted using *P. pygmaeus* as the outgroup. Finally, maximum likelihood branch lengths for the species tree were computed under the GTR+Gamma model and enforcing a molecular clock in PAUP*.

**Rattlesnake data set.** This data set from [9] contained DNA sequence data from 19 genes for 24 individuals of six subspecies of *Sistrurus* rattlesnakes and 2 outgroup species. Each diploid individual is represented by 2 sequences. We first randomly picked 1 sequence per subspecies and 1 outgroup sequence, i.e., 7 sequences in total. The individuals used were the outgroup ac1OUTG (*A. contortrix*), sca151WI (*S. c. catenatus*), sced127AZ (*S. c. edwardsii*), scter115K (*S. c. tergeminus*), smc1NC (*S. m. miliarius*), smi100FL (*S. m. barbouri*), and sms10K (*S. m. streckeri*). As 3 out of the 19 genes did not contain data for all species (LAM did not contain data for scter115K and smi100FL, ef did not contain data for sced127AZ, and fgb did not contain data for ac1OUTG), we subsequently estimated gene trees for only 16 genes under the GTR+Gamma model using RAxML version 8.2.12. Using SVDquartets we then estimated a species tree and computed maximum likelihood branch lengths for the species tree were computed under the GTR+Gamma model and enforcing a molecular clock in PAUP*.

**Rodent data set.** This data set was originally collated by [10] and re-analyzed by [4]. We downloaded the data set from [4] (`https://datadryad.org/stash/dataset/doi:10.5061%2Fdryad.9p8cz8wc5`) and used the data found in the directory `2_Shen_et_al_Empirical_datasets/1_animal/removal_of_inconsistent_genes` consisting of DNA sequence data as well as gene and species tree estimates based on 794 genes for 37 rodent species. More precisely, we used the gene trees provided in the subfolder `ASTRAL`, but discarded 33 of them, which were incomplete, i.e., which did not contain all 37 taxa (the gene trees removed correspond to the following lines in the file `37taxa_794genes_Rodent_ML2ASTRAL_CCC.trees`: 23, 94, 95, 105, 145, 155, 242, 251, 259, 272, 275, 315, 320, 332, 366, 368, 405, 513, 549, 579, 605, 608, 614, 618, 637, 673, 676, 681, 688, 716, 747, 757, 793). Thus, we used a total of 761 gene trees. Moreover, we excluded the corresponding regions from the full

alignment (file `37taxa_794genes_Rodent_ML2ASTRAL_CCC.fas` found in the subfolder `Concatenation_ML`) and estimated a species tree based on the sequence data corresponding to the remaining 761 genes using SVDquartets [11] as implemented in the PAUP* package [12]. Finally, maximum likelihood branch lengths were computed under the GTR+Gamma model and enforcing a molecular clock in PAUP*. Both gene and species trees were rooted using *R. norvegicus* (Murine_Ref_Rat_20161116 (Rat37)) as the outgroup.

**Snake data set.** This data set obtained from [13] (`https://datadryad.org/stash/dataset/doi:10.5061/dryad.rb5nc`) contained DNA sequence data as well as gene and species tree estimates for 31 caenophidians and 2 outgroup species. For our analysis, we used gene and species trees estimated by the authors under a maximum likelihood framework (files `col33_ML.trees` and `col_ML33.tre`). The trees were rooted using species *A. carolinensis* as the outgroup. Finally, maximum likelihood branch lengths were computed under the GTR+Gamma model and enforcing a molecular clock in PAUP*.

**Yeast data set.** This data set from [14] contained DNA sequence data from 106 genes for 8 yeast species. We estimated gene trees under the GTR+Gamma model and a species tree using RAxML version 8.2.12, respectively SVDquartets. In both cases, trees were rooted using species *C. albicans* as the outgroup. Finally, maximum likelihood branch lengths for the species tree were computed under the GTR+Gamma model and enforcing a molecular clock in PAUP*.

# References


[1] McGowen MR. Toward the resolution of an explosive radiation—A multilocus phylogeny of oceanic dolphins (Delphinidae). Molecular Phylogenetics and Evolution. 2011;60(3):345–357. doi:10.1016/j.ympev.2011.05.003.

[2] Stamatakis A. RAxML Version 8: A tool for Phylogenetic Analysis and Post-Analysis of Large Phylogenies. Bioinformatics. 2014;30:1312–1313. doi:10.1093/bioinformatics/btu033.

[3] Steenwyk JL, Opulente DA, Kominek J, Shen XX, Zhou X, Labella AL, et al. Extensive loss of cell-cycle and DNA repair genes in an ancient lineage of bipolar budding yeasts. PLOS Biology. 2019;17(5):e3000255. doi:10.1371/journal.pbio.3000255.

[4] Shen XX, Steenwyk JL, Rokas A. Dissecting Incongruence between Concatenation- and Quartet-Based Approaches in Phylogenomic Data. Systematic Biology. 2021;70(5):997–1014. doi:10.1093/sysbio/syab011.

[5] Song S, Liu L, Edwards SV, Wu S. Resolving conflict in eutherian mammal phylogeny using phylogenomics and the multispecies coalescent model. Proceedings of the National Academy of Sciences. 2012;109(37):14942–14947. doi:10.1073/pnas.1211733109.

[6] Springer MS, Gatesy J. The gene tree delusion. Molecular Phylogenetics and Evolution. 2016;94:1–33. doi:10.1016/j.ympev.2015.07.018.

[7] Boachon B, Buell CR, Crisovan E, Dudareva N, Garcia N, Godden G, et al. Phylogenomic Mining of the Mints Reveals Multiple Mechanisms Contributing to the Evolution of Chemical Diversity in Lamiaceae. Molecular Plant. 2018;11(8):1084–1096. doi:10.1016/j.molp.2018.06.002.

[8] Chen FC, Li WH. Genomic Divergences between Humans and Other Hominoids and the Effective Population Size of the Common Ancestor of Humans and Chimpanzees. American Journal of Human Genetics. 2001;68:444–456. doi:10.1086/318206.

[9] Kubatko LS, Gibbs HL, Bloomquist EW. Inferring Species-Level Phylogenies and Taxonomic Distinctiveness Using Multilocus Data in Sistrurus Rattlesnakes. Systematic Biology. 2011;60(4):393–409. doi:10.1093/sysbio/syr011.

[10] Roycroft EJ, Moussalli A, Rowe KC. Phylogenomics Uncovers Confidence and Conflict in the Rapid Radiation of Australo-Papuan Rodents. Systematic Biology. 2019;69(3):431–444. doi:10.1093/sysbio/syz044.

[11] Chifman J, Kubatko L. Quartet Inference from SNP Data Under the Coalescent Model. Bioinformatics. 2014;30(23):3317–3324. doi:10.1093/bioinformatics/btu530.

[12] Swofford D. PAUP*: Phylogenetic Analysis using Parsimony (*and other methods). wwwpaupphylosolutionscom, version 4a168. 2021;.

[13] Pyron RA, Hendry CR, Chou VM, Lemmon EM, Lemmon AR, Burbrink FT. Effectiveness of phylogenomic data and coalescent species-tree methods for resolving difficult nodes in the phylogeny of advanced snakes (Serpentes: Caenophidia). Molecular Phylogenetics and Evolution. 2014;81:221–231. doi:10.1016/j.ympev.2014.08.023.

[14] Rokas A, Williams BL, King N, Carroll SB. Genome-scale approaches to resolving incongruence in molecular phylogenies. Nature. 2003;425(6960):798–804. doi:10.1038/nature02053.
